# Targeting interleukin-17 receptors

**DOI:** 10.18632/oncotarget.4892

**Published:** 2015-07-17

**Authors:** Wen-Hwa Lee, Heng-Hsiung Wu, Chun-Kai Huang

**Affiliations:** Graduate Institute of Clinical medicine, China Medical University, Taichung, Taiwan

During the last decade, increasing evidence suggests a strong association between chronic inflammation and cancer development among different types of cancer. The dysregulation of pro-inflammatory cytokines or their receptors is a common feature observed in many cancers [1]. Cancer cells take advantage of cytokine or cytokine receptor overexpression to benefit their own growth or invasive capacity via the autocrine or paracrine loop. The improved understanding about the crosstalk between cancer cell and tumor microenvironment may raise the foundations for development of novel drugs in cancer treatment.

**Figure 1:**
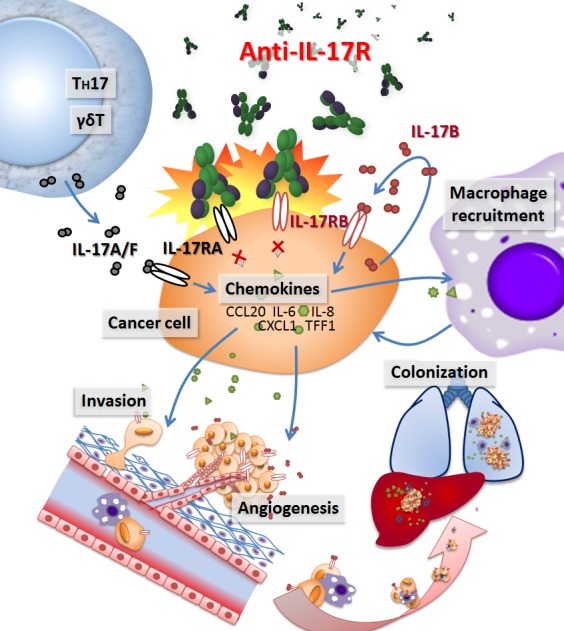
Schematic diagram showing the roles of IL-17 signaling in pancreatic cancer and blockade of the signal by antibodies as a potential treatment

Pancreatic cancer is a malignant diseases associated with significant intra- and peri-tumoral inflammation. Recently, we showed that the pro-inflammatory autocrine/paracrine IL-17B/IL-17RB signaling is essential for pancreatic cancer malignancy [2]. Overexpression of IL-17RB strongly correlates with post-operative metastasis and inversely correlates with progression-free survival in pancreatic cancer patients. The activated IL-17B/IL-17RB signaling pathway increases the tumorigenic and metastatic abilities of pancreatic cancer cells. The expressions of CCL20, CXCL1, IL-8, and TFF1, induced by autocrine/paracrine IL-17B/IL-17RB signaling through ERK1/2 pathway in pancreatic cancer cells, enhance inflammation in the tumor microenvironment via recruiting neutrophils, MQ and lymphocytes, which further support cancer cells survival and facilitate metastasis. Likewise, chemokines induced by IL-17B/IL-17RB may also be secreted from stromal cells and participate in MQ and endothelial cell recruitment to promote pancreatic cancer progression. Except for TFF1 that is predominantly expressed in cancer cells, CCL20, CXCL1 and IL-8 can be detected both in pancreatic cancer cells and tumor surrounding stroma, particularly in inflammatory cells, suggesting a vicious cycle between cancer cells and infiltrating immune cells in promoting tumor malignancy as illustrated in Figure 1. It appears that IL-17B/IL-17RB signaling enhances cancer cell malignancy and simultaneously remodels its microenvironment (i.e. MQ and vasculogenic endothelial cells recruitment) to facilitate metastasis by, in part, secreting these chemokines. Taken together, the IL-17B/IL-17RB signaling not only emerges as an important regulator of pancreatic cancer growth and metastasis, but also serves as an obvious target for pancreatic cancer treatment [2].

To translate this finding into a potential clinical application, a monoclonal antibody recognizing the native form of IL-17RB was generated. Treatment with this newly made monoclonal antibody not only effectively blocks pancreatic tumor metastasis, but also significantly prolongs survivals in a mouse xenograft model. These results suggest that IL-17B/IL17RB signaling is a major contributor to the highly aggressive characteristics of pancreatic cancer, and provide a practical approach to tackle this disease [2]. Similarly, blocking IL-17RB signal reduces breast tumor growth [3]. Thus, targeting IL-17B/IL-17RB is likely a useful approach for treating cancers with this activated pathway.

The presence of other IL-17 members in tumor microenvironment has been reported as a part of the inflammatory conditions that promotes tumorigenesis and metastasis. The IL-17 family consists of six cytokines, IL-17A through IL-17F, with 20-50% sequence homology. IL-17A and IL-17F are pro-inflammatory cytokines exclusively secreted by activated T-cells. IL-17B, IL-17C, IL-17D and IL-17E are expressed in various tissues at low amounts. The cognate receptors for the IL-17 family, IL-17RA to IL-17RE, have been identified, but the physiological roles of these receptors have yet to be fully characterized [4]. Interestingly IL-17A has been shown to promote tumor growth through an IL-6-Stat3 signaling pathway, suggesting that IL-17A paracrine network can also serve as a target for cancer treatment [5].

McAllister and co-workers demonstrated a potential value of IL-17A/IL-17RA blockade in pancreatic intraepithelial neoplasia (PanIN) progression in a murine model. They found that activation of Kras in PanIN cells not only recruited CD4+T and γδT cells to PanIN surrounding stroma to enhance the chronic pancreatitis, but also induced the overexpression of IL-17RA in the PanIN cells. Interestingly, neutralization of IL-17A/IL-17RA pathway via specific antibodies delays the progression of PanINs [6]. Consistently, in skin tumor, the recruitment of IL-17A-producing CD4+T cells was shown to mediate enhancement of papilloma formation, and abrogation of IL-17A signaling with antibody significantly attenuates skin tumor formation [7]. Although it remains to be seen in human tumors, these two studies from murine models suggest that targeting IL-17A/IL-17RA axis can also be a valuable approach for cancer treatment.

In sum, these studies clearly indicate the critical roles of IL-17 signaling in cancer progression and a useful approach for treating cancer by intercepting this signal. However, elucidating the intricacy between cancer cells and its inflammatory microenvironment warrants more efforts from immunologists and cancer biologists.
